# Community readiness for adolescents’ overweight and obesity prevention is low in urban South Africa: a case study

**DOI:** 10.1186/s12889-016-3451-9

**Published:** 2016-08-11

**Authors:** Rebecca Pradeilles, Emily K. Rousham, Shane A. Norris, Joanna M. Kesten, Paula L. Griffiths

**Affiliations:** 1 Faculty of Epidemiology and Population Health, London School of Hygiene and Tropical Medicine, London, UK; 2 Centre for Global Health and Human Development, School of Sport, Exercise and Health Sciences, Loughborough University, Loughborough, UK; 3 MRC/Wits Developmental Pathways for Health Research Unit, University of the Witwatersrand, Johannesburg, South Africa; 4 NIHR Collaboration for Leadership in Applied Health Research and Care West (NIHR CLAHRC West) at University Hospitals Bristol NHS Foundation Trust, Bristol, UK; 5 NIHR Health Protection Research Unit on Evaluation of Interventions, University of Bristol, Bristol, UK; 6 School of Social and Community Medicine, University of Bristol, Bristol, UK

**Keywords:** South Africa, Adolescents, Overweight, Obesity, Church-based interventions, Community readiness, Urban

## Abstract

**Background:**

South Africa is undergoing epidemiological and nutrition transitions with associated increases in the incidence of overweight, obesity and diet-related chronic diseases. With the emergence of the nutrition transition in South Africa, there is an urgent need for interventions to prevent overweight and obesity in children and adolescents as risk factors for chronic diseases in adolescence may track throughout later life. This research explored the potential for faith-based organisations (FBOs) to be used as community organisations for overweight and obesity prevention interventions in adolescents by assessing the readiness of religious leaders to engage in such interventions.

**Methods:**

Surveys and focus group discussions (FGDs) were conducted with 51 religious leaders in Johannesburg and Soweto. The Community Readiness Model (CRM) survey was chosen to determine the stage of readiness of this community regarding overweight and obesity prevention. Six different dimensions were assessed in the CRM (community efforts, knowledge of efforts, leadership, community climate, knowledge of the issue, resources). The surveys were scored according to the CRM protocol. The survey data were supplemented with findings from FGDs. Thematic analysis was used to analyse the FGDs.

**Results:**

The mean community readiness score was 2.57 ± 0.76 which equates with the “denial/resistance stage”. The mean readiness score for resources was the highest of all the dimensions (3.77 ± 0.28), followed by knowledge of the issue (3.20 ± 0.51). The lowest score was seen for community knowledge of efforts (1.77 ± 1.50), followed by community climate (2.00 ± 0.64). FGDs helped interpret the CRM scores. FGDs showed that religious leaders were enthusiastic and recognised that their role was not limited solely to spiritual guidance and mentoring, but also to physical well-being.

**Conclusions:**

Religious leaders recognised that they act as role models within the community and thus have a role to play in improving adolescent health. They have some knowledge about the overweight/obesity issue and some of the resources could be made available to support overweight/obesity prevention-related initiatives. However, the low community knowledge of efforts and the negative prevailing attitude of the community towards overweight and obesity highlight the need to increase awareness of this issue prior to implementing initiatives on overweight and obesity prevention.

**Electronic supplementary material:**

The online version of this article (doi:10.1186/s12889-016-3451-9) contains supplementary material, which is available to authorized users.

## Background

South Africa is undergoing the nutrition transition as evidenced by changes in lifestyle behaviours and the increasing prevalence of overweight and obesity [1–4]. It has one of the highest prevalences of overweight and obesity in Sub-Saharan Africa [5]. The 2012 South African National Health and Nutrition Examination Survey showed that 30.7 % of adult men and 64.0 % of women are overweight or obese [6]. In adolescents aged 15–17 years, the prevalence of overweight and obesity is lower with 8.8 % of males and 27.3 % of females being either overweight or obese [6]. With the emergence of the nutrition transition in South Africa and its implications for health, there is an urgent need for interventions to be implemented to prevent overweight and obesity in adolescents as this group will become the next generation of adults and risk factors for chronic diseases in adolescence may track throughout later life [7].

The need for community involvement in efforts to prevent overweight/obesity and promote healthy eating behaviours has been identified [8]. Minkler et al. [9] state that community-based participatory research (CBPR) stimulates a sense of empowerment and community ownership of the health programme, thus resulting in improved participation and long-term sustainability. However, communities are at different stages of readiness for intervention and this is known to be a fundamental determinant for the success of implemented interventions [10]. Therefore, interventions that aim to incorporate the community must make an initial assessment of its readiness.

The CRM is a model “that integrates a community’s culture, resources, and level of readiness” (p3) [11] in order to approach existing problems in the community. It serves as a theoretical framework for understanding and improving community readiness [10]. The term readiness is defined as “the degree to which a community is prepared to take action on an issue” (p3) [11] whilst also referring to the observable and psychological factors that affect the ability of a community to change [12, 13]. The CRM development has been described in detail elsewhere [14]. Community readiness can be measured through in-depth individual interviews [15] or via a survey [12] conducted with key informants in the community (i.e., community members who work or have a leadership role and who are knowledgeable about the issue being examined). As in-depth interviews are time and resource intensive for participants and researchers [12], a survey can be more widely applied, particularly during pilot work. However, qualitative analysis of responses is essential to support the quantitative scoring and it is not recommended to use one without the other [16]. Very little is known about the application of the CRM in low- and middle-income countries (LMICs), with a very small number of studies having used this tool to assess community readiness, for example, for human immunodeficiency virus (HIV)/acquired immune deficiency syndrome (AIDS) interventions in rural Bangladesh [17] and Liberia [18] and for the study of disability in India [19]. The CRM has been applied to childhood obesity prevention in the USA [20–22], Australia [23] and in the UK [24]. However, no evidence regarding the CRM being applied to childhood obesity prevention currently exists in LMICs. This provides further support for a mixed-methods approach to enable the qualitative responses to help interpret the meaning of the quantitative readiness scores.

Churches (FBOs) are increasingly recognised as being popular settings for implementing health promotion programmes [25–28]. A review by DeHaven et al. [29] on studies published between 1990 and 2000 identified 53 church-based health promotion programmes implemented in the USA. The focus of the programmes was mainly on health issues such as cardiovascular diseases, cancer, smoking cessation, nutrition (fruit and vegetable intake) and weight control. Fewer studies have investigated the role of churches in addressing childhood obesity prevention [30, 31]. He et al. [31], conducted interview discussions with Latino church leaders in Texas, US, in order to obtain their views on childhood obesity prevention. The Church as a cultural institution is unique in its ability to reach individuals, families and people of all ages and socio-economic backgrounds. Alongside the obvious religious services provided, religious organisations also offer social, organisational, and health services [28, 32]. The Church setting also offers an opportunity to expose people to health promotion messages regularly. The pastor and other religious leaders have a pivotal role in the community and act as role models to their congregation. This is especially the case in African American churches, with the pastor considered “the guiding force” [33].

Previous qualitative work conducted with adolescents in the South African urban context has identified the Church as being an important part of an adolescent’s community [34]. Furthermore, 69.1 % of the sample of 18 year old adolescents in the Birth to Twenty cohort reported belonging to a religious group [35]. Of these, the majority were Christian (88.6 %). In terms of the significance religion plays in the lives of these adolescents, 39.5 % reported religion as important and 51.1 % as very important. In the past six months, 61.1 % reported attending ordinary weekly services (6.1 % occasionally, 22.7 % sometimes and 32.3 % weekly). These findings show the importance of the Church and religion in adolescents’ lives and suggest that the Church community might therefore be a useful setting for intervention in the South African context.

There is however a need to understand the readiness of the Church community and other religious communities within the South African context as vehicles for intervention on overweight and obesity as no evidence from South Africa currently exists. Therefore, the aim of this study is to explore the potential for religious groups such as Churches to act as community-based organisations for overweight and obesity prevention interventions, by assessing the readiness of religious leaders to engage in such interventions.

## Methods

### Study design

In order to answer our research question, we used a mixed methods design, combining both the CRM survey and FGDs. The CRM survey was used to generate the community readiness scores and answer the question as to how ready this community is for obesity interventions. The FGDs complemented the survey by providing an in-depth interpretation of the scores achieved and answered the question as to why the community gave these scores as well as helping to understand what might be appropriate target points for future interventions. The rationale for using this mixed methods approach rather than face-to-face interviewing was pragmatic due to the resource intensive nature of individual interviews, the limited availability of the participants and time available in the field for this initial study. Similarly to in-depth interviews, FGDs can elicit in-depth opinions and experiences. They also allow gathering perspectives on a specific topic from numerous people simultaneously and generate greater breadth of information because people can build on each other’s responses. This mixed methods design allowed us to obtain views from a much greater number of people in a relatively short period of time in a resource-poor setting. By using a combination of methods (survey and FGDs) and thus a novel application of the tool we intended to produce an in-depth assessment of readiness.

### Study setting

This study was conducted in Johannesburg-Soweto, the largest urban area in South Africa which has a population of 4.4 million [36], with a relatively high Human Development Index [37] alongside extreme inequalities and issues. Indeed, in Johannesburg-Soweto, there are problems of poverty, unemployment, violence, high prevalence of HIV/AIDS, chronic diseases and food insecurity [38].

### Data collection

The community of interest in this study was the Church and the religious leaders were the key informants interviewed. Plested et al. ([11]:p6) defines a community as a “geographical area, a group within that area, an organisation, or any other type of identifiable “community””. Christian Churches were recruited in the area of Johannesburg-Soweto using information letters, telephone calls and a system of snowball sampling. Churches were initially identified via staff members working on the Birth to Twenty Plus cohort study (Bt20+) at the Witwatersrand University in the area of Johannesburg-Soweto. Staff members who were approached were active members of the Church and attended the services on a regular basis. Bt20+ staff introduced the lead researcher to the pastors of the different Churches and facilitated communication between the two parties. Prior to data collection, the main researcher visited the targeted churches and approached the pastor to discuss potential study participation. The inclusion criteria for study participants were: 1) those who were affiliated with the Church, 2) those who had a leadership role in the congregation (teachers, coaches, pastors, bishops, elders), 3) those who could participate in one of the scheduled FGDs and 4) those who were also comfortable communicating in English (the language of the researcher). If study participants agreed to participate, they were given a participant information letter and written informed consent was taken on the day of the data collection. Data collection (administration of the CRM survey (n = 51) and focus groups (n = 6)) was conducted from November 2012 to January 2013 with a sample of predominantly black South African religious leaders, including pastors, bishops, teachers, and elders. Data were collected from four churches located in relatively poor/medium socio-economic status (SES) neighbourhoods and two churches located in relatively wealthy neighbourhoods. From the eight churches that were approached, seven were willing to participate in the study. Interviewing was stopped after six FGDs as saturation of ideas had been reached, therefore the 7th church was not used [39, 40].

#### CRM survey

The CRM survey was adapted from the CRM questionnaire [11] to suit the aim of this particular study as recommended by the authors who developed the tools. The survey assessed six different dimensions of readiness: community efforts, knowledge of efforts, leadership, community climate, knowledge of the issue, resources. For this study the language in the CRM survey was simplified (i.e., use of an appropriate terminology given the literacy levels of the community of interest) as a result of piloting. Based on the findings of these pilot tests, the tool was amended and adapted to the urban South African context (re-phrasing of sentences, altering terminology and modifying the tool’s Likert scale). The Likert scales used in the survey to measure opinions were challenging for the participants to comprehend, therefore a culturally adapted example was used at the beginning of the survey to facilitate the understanding of the scale system. Smiley faces were also added to the scales in order to aid comprehension for a population that has lower literacy levels. Participants with a lower level of education found the CRM survey challenging to complete. The number of questions included in the survey was also limited to those needed for scoring readiness as specified by the CRM authors [11].

Participants completed the CRM survey consisting of 37 semi-structured questions before the FGD (see Additional file 1). Participants were asked to complete written responses to these questions. Background socio-demographic information was provided by participants via self-completion questions at the beginning of the survey.

#### FGDs

After completion of the CRM survey, the FGDs were performed. Open-ended questions used in the FGDs addressed community issues; people’s social perceptions of the body, perceived determinants of obesity, the role of the Church in adolescents’ health and the nature of the relationship between religious leaders and adolescents (see Additional file 2). The number of participants in each FGD ranged from 4 to 12 (n = 51) and comprised both males and females. FGDs lasted between 60 and 90 min. In addition to the main researcher (RP) who led the FGDs, a local research assistant was present to take notes and to provide clarification at the end of the FGDs on any terminology not understood by the main researcher. Participants were provided with healthy snacks and drinks during the FGDs.

### Data analysis

#### CRM survey

Descriptive statistics were performed on socio-demographic characteristics using IBM SPSS (version 19). The surveys were scored according to the CRM protocol explained in the CRM handbook [11]. Initially, each survey was read through in full in order to get a general idea of the content. Following that, the scorers compared each response to the nine anchored rating statements for the dimension being assessed and matched it to the most appropriate anchored rating statements [11]. After scoring, the average score for each dimension was calculated and a score for each Church then calculated as the sum of average scores for each dimension divided by the number of dimensions. Finally, an overall stage of community readiness for all churches combined was calculated as the sum of scores for each church divided by the number of churches included in the study (n = 6) (range 1–9). Scoring CRM surveys using pre-defined anchored rating statements has been criticised [12, 41] for permitting too much researcher subjectivity into the process. In order to reduce this potential bias, the CRM surveys were scored independently (RP and JK) by two reviewers and then combined. If discrepancies arose between reviewers, these were discussed until consensus was reached.

#### FGDs

The audio recordings from FGDs were transcribed verbatim by a local trained transcriber, anonymised and reviewed by the researcher (RP) for accuracy. The transcripts were analysed using thematic analysis which consists of identifying repeated patterns of meaning [42]. After familiarising with the data, the researcher systematically coded the transcripts line by line and generated initial codes. The initial codes were used to produce themes by searching for frequently occurring and important concepts relating to the research question. Themes consisting of grouped codes were then defined in a codebook which summarised their meanings (see Additional file 3). One coded transcript out of the six FGDs was reviewed and checked by a second coder. The coders then agreed on the final code definition and rules for its use. The thematic analysis of the FGDs led to the identification of numerous themes and sub-themes. However, for this study, as we were interested in understanding the community readiness scores achieved, we decided to focus on certain themes that emerged from the FGDs to triangulate with the quantitative findings.

## Results

### Socio-demographic characteristics of the sample

Table 1 shows that the sample (n = 51) was relatively young with 60.7 % aged 20–39 years old. More than half of the sample had completed a higher education degree (53.0 %). An almost equal proportion of participants were single (45.1 %) or married (47.1 %). Most commonly, the leaders who attended the FGDs were either pastors (26.0 %) or youth leaders (32.0 %) such as youth pastor, sports coach, etc. The other leaders in the sample were teachers, elders and other members of the Church. More than two thirds of the sample had been associated with the Church for more than ten years.

**Table 1:** Socio-demographic characteristics of religious leaders

	Overall	
	n	%
Sex		
Male	32	62.8
Female	19	37.2
Population group		
White	4	7.8
Black African	46	90.2
Mixed ancestry	1	2
Age		
< 20 years old	1	2
20-29 years old	15	29.4
30-39 years old	16	31.3
40-49 years old	10	19.6
50-59 years old	8	15.7
≥ 60 years old	1	2
Level of education		
Primary school completed	2	3.9
Secondary school completed	5	9.8
Some high school	4	7.8
High school completed	13	25.5
More than high school	27	53
Marital status		
Single	23	45.1
Married	24	47.1
Widow	2	3.9
Divorced	2	3.9
Cohabiting	_	_
Role in the institution		
Pastor	13	26
Teacher	5	10
Elder	8	16
Health professional	_	_
Youth worker/youth leader	16	32
Other	8	16
How long have you been associated with the church		
Less than a year	2	4
1-5 years	7	14
5-10 years	8	16
More than 10 years	33	66

### Community readiness scores

The mean readiness score was 2.57 ± 0.76 (Table 2). This corresponds to the second lowest of nine stages of the community readiness. This stage is called the “denial/resistance stage” and is reached when “at least some community members recognise that the issue is a concern, but there is little recognition that it might be occurring locally” (p9) [11].

**Table 2:** Community readiness scores obtained from a sample of religious leaders (n = 51)

Dimensions	Community efforts	Knowledge of community efforts	Leadership	Community climate	Knowledge of the issue	Resources
Mean score (SD)	2.30 (0.92)	1.77 (0.50)	2.41 (0.78)	2.00 (0.64)	3.20 (0.51)	3.77 (0.28)
Overall readiness stage (SD)	2.57 (0.76)	(Denial/resistance stage)				

**Fig. 1:**
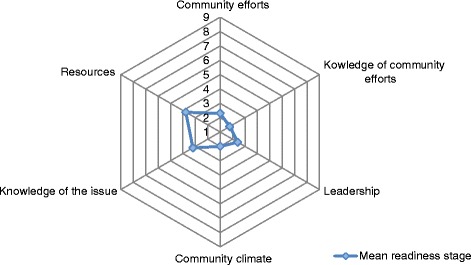
Community readiness assessment graph showing the average score per dimension for participants from six churches

The following section focuses on interpreting scores within each dimension (Fig. 1) by using relevant themes from the FGDs:

#### Community efforts and knowledge of efforts

The scores for community efforts (i.e., programmes and policies in place that address the issue) and knowledge of efforts (i.e., the extent to which community members know about local efforts and effectiveness) were relatively low (2.30 and 1.77 respectively). These results suggest, in accordance with the CRM handbook [11], that: 1) no efforts currently exist to address the obesity issue; 2) the community has no knowledge of the need for efforts to address the issue.

The following themes from the FGDs were used to interpret the community efforts and knowledge of efforts scores: community issues (i.e., social, health and safety issues), resources and church’s role in adolescent health. The low scores can be explained by the fact that religious leaders recognised overweight or obesity as a problem among adults and in the wider population, but did not view it as a concern among adolescents in their congregation. Furthermore, in the FGDs adolescents were described as experiencing various problems in their daily lives (social and health issues) that have greater priority.

*“In our Church, we have challenges like adolescents being raped, physically abused, homeless and poverty stricken, and obesity has in a way been shoved low on the list of priorities relatively.”*

Female leader, Evangelical Church, Johannesburg suburb

The lack of resources and challenges faced by the religious leaders could also explain the low scores for community efforts and community knowledge of efforts. Some members reported that resources were lacking (information, space and facilities, time, money, and people) and also made mention of perceptions as a barrier for the development of community efforts for the obesity issue. Spiritual satisfaction over physical satisfaction was also discussed.

*“…I think that we do have the structures that can plan and make the resources available but such issues are not priorities within the Church because most people just focus on satisfying their spirituality side of things and the physical being is ignored. Cleanliness and healthiness are in line with Godly standards. We just need to identify the needs and plans accordingly.”*

Male leader, Methodist Church, Soweto

Another reason for not having obesity programmes could be the stigmatisation associated with it.

*“We wouldn’t of course have a programme for obese people as that would stigmatise them.”*

Female leader, Congregational Church, Soweto

*“Yes some people are big but it has never been viewed as a problem, we do not discriminate against big people or make them feel uncomfortable in any way.”*

Female leader, Methodist Church, Soweto

#### Leadership and community climate

The scores for leadership (i.e., the extent to which appointed leaders and influential community members are supportive of the issue) and community climate (i.e., the prevailing attitude of the community towards the issue) were relatively low (2.41 and 2.00 respectively). These results suggest, in accordance with the CRM handbook [11], that: 1) the leadership believes this is not an issue in their community; 2) the prevailing attitude of the community towards the issue is not one of responsibility and empowerment. The following themes from the FGDs were used to interpret the leadership and community climate scores: church’s role in adolescents’ life and relationship between leaders and adolescents. Despite the scores being low, a strong sense of responsibility towards adolescents’ health and well-being in general was apparent in the FGDs. A leader from the Evangelical Church in Johannesburg stated that *“mostly Church is about spiritual growth and not personal health and hygiene really.”* However, leaders from other Churches agreed that their role goes beyond the spiritual guidance and mentoring. They also play a role in physical well-being.

*“We are responsible for the well-being of the young people that attend at this Church, their health is part of our responsibility towards them (…).*

Male leader, Methodist Church, Johannesburg

*“The Church plays a very important role in this regard as it has always been a central meeting place for people of different backgrounds coming together for one agenda. The Church is responsible for uplifting and informing people spiritually and otherwise. “*

Female leader, Roman Catholic Church, Soweto

Religious leaders described themselves as role models for adolescents but the youth leaders (youth pastor, youth coach, etc.) were recognised as the most influential people working with adolescent members of the congregation.

*“…these kids do look up to us as their role models and if we lead by example then there is a big chance that the next generation after us will be conscious of healthy living options and issues like obesity could be history.”*

Male leader, Methodist Church, Soweto

#### Knowledge of the issue of obesity

The readiness score for knowledge of the issue (i.e., the extent to which community members know about the causes and consequences of the issue) was low but one of the highest of all the dimensions (3.20). This result suggests, in accordance with the CRM handbook [11], that a few people in the community have some knowledge of the obesity issue. Leaders’ perceptions about the causes of obesity ranged from individual, household, organisation, neighbourhood and national levels. The following themes from the FGDs were used to interpret the scores for knowledge of the issue of obesity: unhealthy dietary and physical activity patterns.

At the individual level, leaders reported lifestyle factors (unhealthy dietary and physical activity patterns) to be the main drivers of obesity.

*“It could be one’s diet. A lot of doctors and nutritionists have detected a new trend amongst black people since they have financially developed. They can now afford takeaways like McDonald’s and a lot of junk food.”*

Male leader, Methodist Church, Soweto

*“One of the major causes of obesity is lack of exercise.”*

Male leader, Methodist Church, Johannesburg

*“It is easier to socialise on the phone than it is to socialise outside. Now we have all the gadgets to keep us entertained and connected to our peers without actually having to go outside to play and interact.”*

Female leader, Roman Catholic Church, Soweto

Individual level causes such as “laziness”, “lack of fasting”, “genetics”, “stress, depression and emotions”, “medication or medical condition” were also mentioned. Body size perception was seen as a factor favouring overweight and obesity.

*“Perception could also be a major challenge. As Africans we believe that bigger is always better.”*

Male leader, Methodist Church, Soweto

At the household level, the main factors that were discussed were affordability and cultural change regarding family dynamics (work life balance, working women, etc.). These factors were related to dietary patterns.

*“…Bad foods are cheap and easily accessible. The good stuff and the organic foods are expensive. The good quality food that we ate before has now become out of reach and very expensive and this poses a major challenge for us when we have to choose what to eat. Everything has been refined today and the quality food has been compromised. Fruit has been a luxury and not an everyday readily available commodity. “*

Female leader, Congregational Church, Soweto

*“Life is now fast paced and both parents are working and as a result nobody has the time to prepare a healthy balanced meal for the family and unhealthy eating may cause obesity, heart diseases, and hypertension as a result which were never an issue for adolescents in my time. “*

Male leader, Pentecostal Church, Johannesburg

At the neighbourhood and society levels, leaders discussed the role of the government in promoting unhealthy food environments and highlighted the lack of development programmes, facilities for adolescents to engage in physical activity and information regarding healthy eating.

*“One of the major causes of obesity is lack of exercise and that could also be fuelled by lack of platforms or resources for young people to embark on exercise or even be interested in sports or exercise.”*

Male leader, Methodist Church, Johannesburg

*“… There are no educational programmes in place to teach people on ways to curb obesity.”*

Male leader, Congregational Church, Soweto

*“…The government is playing a major role in promoting obesity because they allow companies to distribute foods that cause obesity. There are no outreach programmes in place by the government to teach people on obesity. The government is basically to blame.”*

Male leader, Congregational Church, Soweto

*“It is also because of lack of information as well on what people should eat and how much of it they should eat. “*

Female leader, Roman Catholic Church, Soweto

#### Resources

The readiness score for resources (i.e., the extent to which people, time, money and space are available to support efforts) was low but the highest of all the dimensions (3.77). This finding suggests that the community is not sure where the resources would come from to initiate efforts [11]. Availability of resources differed between the different Churches. The following themes from the FGDs were used to interpret the resources scores: people, time, money, space/facilities and religious writings. Leaders recognised that people within the Church could be used as a resource for obesity prevention/health promotion initiatives.

*“I think that we do have people within the congregation that are expert in the various fields that are necessary in helping the adolescents and all that we need to do is to initiate the move. Any other information can be outsourced. We can always grab an external person to be a guest speaker.”*

Female leader, Methodist Church, Soweto

*“We have teachers, social workers and nurses amongst others within the Church”.*

Female leader, Methodist Church, Soweto

Leaders also indicated that they have potentially good networks beyond the immediate church community.

*“We operate as a youth body in a community within a bigger community. There are various activities like sports and others that we are involved in and there are other networks and resources within the bigger circle”.*

Female leader, Methodist Church, Johannesburg

Another important resource discussed by leaders was the access to adolescents.

*“The fact that we have these kids all in one place is a resource on its own”*

Male leader, Pentecostal Church, Johannesburg

Time seemed to be a constraint across the six different Churches, from the members’ perspective and leaders’ perspective. However, some leaders were more positive about it.

*“Time is the scarcest of all resources in this community. It hinders most development”.*

Male leader, Methodist Church, Johannesburg

*“With proper planning time can be made. We have a good understanding and credibility with the parents and the idea of kids putting in extra time at Church would be most welcome…”*

Male leader, Pentecostal Church, Johannesburg

Money was not extensively discussed by leaders. It was mainly a problem in churches located in more deprived areas.

*“We normally lack funds to do most projects and we have always had to raise funds for all previous functions”*

Male leader, Methodist Church, Soweto

There were mixed messages towards the availability of facilities and space, depending on the type of church.

*“The space is also a limiting factor when it comes to outdoor activities and exercising…”*

Male leader, Roman Catholic Church, Soweto

*“I think we do have the structures that can plan and make the resources available”*

Male leader, Methodist Church, Soweto

Religious writings were mentioned as a resource that could potentially be used to promote healthy lifestyles in relation to diet more than physical activity.

*“The word of God actually guides us to take care of our bodies.”*

Male leader, Pentecostal Church, Johannesburg

*“Over and above the mention of your body being a temple, the bible makes reference to gluttony. It condemns over eating and gives a stern warning that we shouldn’t make the stomach our god. We are encouraged to eat to live rather than to live to eat.”*

Female leader, Methodist Church, Johannesburg

This resource was seen as valuable when teaching adolescents about healthy lifestyles as *“adolescents pay more attention when there is biblical backing on a principle”* (Male leader, Pentecostal Church, Johannesburg).

## Discussion

The purpose of this research was to evaluate the potential for FBOs to be used as community-based organisations for obesity prevention interventions. This aim was achieved by assessing the readiness of religious leaders to engage in such interventions.

No other studies in Sub-Saharan Africa have used the CRM to assess the stage of community readiness to address overweight/obesity in adolescents, making relevant comparisons difficult. However, studies utilising the CRM for obesity prevention interventions have been conducted in high income countries (HICs) [20–24].

In the present study, the readiness score for resources was the highest of all of the dimensions (3.77), followed by knowledge of the issue (3.20). The lowest scoring dimension was community knowledge of efforts (1.77), with the second lowest score being community climate (2.00). Our results suggest that religious leaders in some churches are relatively knowledgeable about the issue and that there might be potential for resources to be developed to support obesity prevention. The community knowledge of efforts and community climate score being low implies that the awareness of the issue and the need to develop initiatives have to be increased. It is important to mention that the overall readiness score masks some differences between churches. The scores for awareness of overweight and obesity ranged from ‘1. No awareness’ to ‘3. Vague awareness’ demonstrating that not all church communities lacked awareness.

Findings from our study highlight that the community may not be ready to tackle adolescent obesity. The mean obesity readiness score in our study was 2.57 ± 0.76 (“denial/resistance stage”). An overall community readiness score of 2.57 indicates that interventions for this stage should focus on raising awareness that the problem of overweight/obesity exists in this community and address stigma associated with it [11]. In this context, it is important not to single out people who are obese for interventions as this may be experienced as stigmatising.

The scores found in the current study are low compared to those observed in HICs. For example, a UK study of pre-adolescent girls assessed the community’s readiness for implementing both physical activity and dietary interventions to prevent overweight/obesity [24]. The readiness score for the physical activity was 6.08 which corresponds to the ‘initiation stage’ (described by Plested et al. ([11] (p9) as “enough information is available to justify efforts. Activities are underway”). The readiness score for dietary intervention was 5.74, corresponding to the ‘preparation stage’, described as “Active leaders begin planning in earnest. Community offers modest supports of efforts” (p9) [11]. However, the obesity prevention readiness score observed in youth living in a disadvantaged community of a HIC (Latino community in Nebraska) was 3 [22], corresponding to the “vague awareness stage” which is achieved when “most feel that there is a local concern, but there is no immediate motivation to do anything about it” (p9) [11]. This score from a more deprived community is more similar to the stage of readiness observed in the present study (2.57).

Plested et al. [11], in their Community Readiness Handbook, state that for an intervention to be effective, each dimension should be at an equal stage of readiness. Therefore, based on our results, an initial focus on preparation for intervention should be on increasing the community efforts, knowledge of efforts and the community climate. The study has highlighted that the first starting point for an intervention in this setting will be to mobilise communities.

The FGDs which complemented the CRM survey scoped out the potential for churches to be used as settings for obesity prevention interventions in adolescents. Participants in our study felt that FBOs were a good context for intervention delivery in this setting. Indeed, community leaders were enthusiastic and recognised that they were perceived as role models within the community and thus play an important role in improving children and adolescent’s health. The youth leaders (youth pastor, youth coach, etc.) were recognised as the most influential people working with adolescent members of the congregation. Furthermore, religious leaders also recognised that their role was not limited solely to spiritual guidance and mentoring, but also to physical well-being. They mentioned that the scriptures could be used to develop health messages and felt that this was part of their remit. They were also shown to be knowledgeable with regard to the causes and consequences of the obesity issue and highlighted that they could develop the necessary resources (human or physical) to implement obesity prevention interventions.

Several church-based obesity and chronic disease prevention interventions have been conducted in African Americans adults which have focused on: increasing healthy eating patterns (increasing fruit and veg [43, 44]; increasing fruit and veg whilst reducing fat intake [45]; increasing physical activity [46, 47] or increasing both healthy eating patterns and physical activity [48–51]. The positive results of these studies suggest that FBOs have the potential to provide delivery platforms for interventions targeted at preventing obesity. Importantly, all of these interventions emphasised the role that religion and faith can play in influencing behaviour change and improving health.

This study has some limitations. Adolescents’ views on the use of churches as settings for overweight and obesity prevention interventions were not included. This information is essential when designing interventions in adolescents. This was not possible due to very limited time and resources during fieldwork. The lack of adolescent interviews meant we were unable to obtain information about peer influences on diet, physical activity and nutritional status in the FGDs. It is important to mention that the overall community readiness is context specific and the qualitative findings are also likely to be somewhat limited in their transferability to other contexts. Furthermore, as this study collected data from a small sample of churches from within a large metropolitan region, the CRM scores may not be representative of the Johannesburg-Soweto area as a whole. However, the churches covered a range of socio-demographic and geographical areas of the city, that are likely to reflect the types of churches that community members attend. To further reduce this potential limitation, sampling was continued until theoretical saturation was achieved. The lead researcher engaged in a reflective process whilst administering the FGDs, listening to the audio-recordings of the FGDs, reading the transcripts, and taking field notes following the FGDs to begin to identify themes. This reflective process and familiarisation with the data allowed the researcher to get an idea of theoretical saturation of concepts and ideas. However, it is acknowledged that this may be subject to error as it is dependent on a single researcher’s interpretation. Additionally, the CRM tool captures a snapshot of the readiness of the community only during the interview period and is not representative of communities which are continuously changing or evolving [21]. Furthermore, the model places complete responsibility for health issues on the shoulders of communities which as highlighted by the FGDs neglects the importance of societal influences such as the government. A further limitation is linked to the assumption that communities will fit into one of the nine stages of readiness defined [41], whereas in reality, they may be operating at an intermediary stage. Finally, interventions through FBOs might miss the adolescents who are not affiliated with any religious institutions (approximately 30 % in the Birth to Twenty study [35]). However, in this context using other commonly targeted adolescent communities from HIC studies such as schools for intervention, misses the most vulnerable and disadvantaged part of the population in South Africa because school enrolment is not compulsory at this age [52]. This has resulted in a non-universal attendance at school across demographic groups [53]. Government schools in South Africa also operate on more limited resources with larger class sizes [54] making intervention through schools more challenging.

This study is novel in that it is the first to use the CRM to assess the readiness of religious leaders to engage in overweight/obesity prevention interventions in adolescents living in Africa. A further strength of this study is the novel application of the community readiness model, with the use of FGDs in partnership with the CRM survey. This allowed for an in-depth investigation of the scores and why such scores were achieved. The FGDs provide an understanding of the situation within the community and highlight vital information which may aid the design and implementation of interventions for adolescents in urban South Africa. The CRM enables community members, in this instance religious leaders, to be the actors in the process of ameliorating the well-being of fellow members, in this instance adolescents.

## Conclusions

Given the high prevalence of overweight and obesity in adults in South Africa, there is an urgent need for interventions to be implemented to prevent overweight and obesity in adolescents as this group will become the next generation of adults.

This study highlighted that religious leaders in the Church setting could be used as a leverage point in addressing overweight and obesity prevention in adolescents living in urban South Africa. Religious leaders recognised that they act as role models within the community and thus have a role to play in improving adolescent health. Future obesity prevention interventions could potentially be implemented by youth leaders given their influence. Religious leaders have some knowledge about the overweight/obesity issue and some of the resources could be made available to support overweight/obesity prevention-related initiatives. However, the low community knowledge of efforts and the negative prevailing attitude of the community towards overweight and obesity highlight the need to increase awareness of this issue prior to implementing initiatives on overweight and obesity prevention. Overall, this study showed that despite the readiness level being low, the Church community is ready to move forward with the obesity prevention agenda in adolescents. The first actions should aim to increase the awareness of the obesity issue amongst religious leaders and there should be a re-evaluation of the CRM stage after such an intervention. It is also essential to assess the readiness of adolescents prior to the implementation of overweight and obesity prevention interventions.

## Abbreviations

AIDS: acquired immune deficiency syndrome; CBPR: community-based participatory research; CRM: Community Readiness Model; FBOs: faith-based organisations; FGDs: focus group discussions; HICs: high income countries; HIV: human immunodeficiency virus; LMICs: low- and middle-income countries

## Additional files

Additional file 1: The Community Readiness Model survey. (DOCX 26 kb) Additional file 2: Focus group interview guide. (DOCX 226 kb) Additional file 3: Results of the thematic analysis. (DOCX 17 kb)
