# Supplementary material for: Community readiness for adolescents’ overweight and obesity prevention is low in urban South Africa: a case study
Source: BMC Public Health. 2016 Aug 11;16:763. doi: 10.1186/s12889-016-3451-9 (PMC4982405; doi:10.1186/s12889-016-3451-9)
Supplement: Additional file 1: — The Community Readiness Model survey. (DOCX 26 kb) [file 12889_2016_3451_MOESM1_ESM.docx]

**Exploration of the potential to develop obesity prevention interventions for adolescents through religious institutions**

**1. Sex**

^1.^ 🞎 Male ^2.^ 🞎 Female

**2. Age**

^1.^ 🞎 Under 20 years old ^4.^ 🞎 40-49 years old

^2.^ 🞎 20-29 years old ^5.^ 🞎 50-59 years old

^3.^ 🞎 30-39 years old ^6.^ 🞎 Above 60 years old

**3. What is your level of education?**

^1.^ 🞎 Never went to school ^5.^ 🞎 Secondary school completed

^2.^ 🞎 Some Primary school ^6.^ 🞎 Some High school

^3.^ 🞎 Primary school completed ^7.^ 🞎 High school completed

^4.^ 🞎 Some secondary school ^8.^ 🞎 More than high school

**4. What is your marital status?**

^1.^ 🞎 Single ^4.^ 🞎 Divorced

^2.^ 🞎 Married ^5.^ 🞎 Cohabiting

^3.^ 🞎 Widow

**5. What is your role in this Church?**

^1.^ 🞎 Pastor

^2.^ 🞎 Teacher

^3.^ 🞎 Elder

^4.^ 🞎 Health professional

^5.^ 🞎 Youth worker

^6.^ 🞎 Other

If other, specify_______________________

**6. How long have you been associated with this Church?**

^1.^ 🞎 Less than 1 year ^3.^ 🞎 5-10 years

^2.^ 🞎 1-5 years ^4.^ 🞎 More than 10 years

***In many questions in this survey I am going to ask you to respond to questions in relation to a scale. The scale will be from one to ten and you will need to respond according to how much you agree with that particular question.***

***For example, a question might ask: How much do you like Bunny Chow? You will need to respond on the scale from one to ten. If you very much like Bunny Chow, then you would respond “10”. But if you don’t like Bunny Chow at all you would respond with a “1”. If you like it a little bit then you would respond with a “3” or if you somewhat like it then you would respond with “5” or “6” and if you like it you would respond “7” or “8”.***

1 🞎 2 🞎 3 🞎 4 🞎 5 🞎 6 🞎 7 🞎 8 🞎 9 🞎 10 🞎

Not at all Somewhat Very much

**7. How much of a concern is overweight or obesity in adolescents in your congregation? (with 1 being “not at all” and 10 being “a very great concern”)?**

1 🞎 2 🞎 3 🞎 4 🞎 5 🞎 6 🞎 7 🞎 8 🞎 9 🞎 10 🞎

Not at all Somewhat of a concern A very great concern

**8a. Please list the programmes that are available in your congregation to address overweight or obesity?**

^1.^_____________________________________________________________________

^2.^ _____________________________________________________________________

^3.^ _____________________________________________________________________

^4.^ _____________________________________________________________________

**/!\ If no programmes are available, move to question 16 /!\**

**8b. Are any of the programmes you listed in question 8a for adolescents?**

^1.^ 🞎 Yes ^2.^ 🞎 No

**9. Do these programmes you listed in question 8a serve people outside the congregation?**

^1.^ 🞎 Yes ^2.^ 🞎 No

**10. For how many years has your congregation provided overweight or obesity prevention programmes for adolescents?**

^1.^ 🞎 Less than one year ^3.^ 🞎 4-10 years

^2.^ 🞎 1-3 years ^4.^ 🞎 More than 10 years

**11a. How many adolescents from your congregation participate in these programmes that focus on overweight or obesity?**

^1.^ 🞎 Less than 10 ^3.^ 🞎 20-30

^2.^ 🞎 10-20 ^4.^ 🞎 More than 30

**11b. How many adolescents from outside your congregation participate in these programmes that focus on overweight or obesity?**

^1.^ 🞎 Less than 10 ^3.^ 🞎 20-30

^2.^ 🞎 10-20 ^4.^ 🞎 More than 30

**12. List 3 good points of these programmes that focus on preventing overweight or obesity among adolescents:**

^1.^_____________________________________________________________________

^2.^ _____________________________________________________________________

^3.^ _____________________________________________________________________

**13. List 3 weak points of these programmes that focus on preventing overweight or obesity among adolescents:**

^1.^_____________________________________________________________________

^2.^ _____________________________________________________________________

^3.^ _____________________________________________________________________

**14. Do you think these programmes that teach about overweight or obesity have influenced adolescent behaviours?**

^1.^ 🞎 Yes

^2.^ 🞎 No

^3.^ 🞎 Don’t know

**15. Do adolescents in your congregation know about these programmes that teach about overweight or obesity?** (tick one box below)

^1.^ 🞎 No adolescents know about them

^2.^ 🞎 Not very many adolescents know about them

^3.^ 🞎 Some adolescents know about them

^4.^ 🞎 Most adolescents know about them

^5.^ 🞎 All adolescents know about them

^6.^ 🞎 Don’t know

**16. How much of a concern is adults’ overweight or obesity to the leadership in your congregation (tick one box, with 1 being “not at all” and 10 being “of a great concern”)?**

1 🞎 2 🞎 3 🞎 4 🞎 5 🞎 6 🞎 7 🞎 8 🞎 9 🞎 10 🞎

Not at all Somewhat of a concern A very great concern

**17. How much of a concern is adolescents’ overweight or obesity to the leadership in your congregation (tick one box, with 1 being “not at all” and 10 being “of a great concern”)?**

1 🞎 2 🞎 3 🞎 4 🞎 5 🞎 6 🞎 7 🞎 8 🞎 9 🞎 10 🞎

Not at all Somewhat of a concern A very great concern

**18. Are leaders involved in programmes regarding adolescents’ overweight or obesity?**

^1.^ 🞎 Yes ^2.^ 🞎 No

If yes, please explain______________________________________________________

**19. Would the leadership support additional programmes in adolescent health?**

^1.^ 🞎 Yes ^2.^ 🞎 No

If yes, please explain______________________________________________________

**/!\ If your congregation has any programmes that address overweight or obesity please refer to question 20 and 21, if not, then go to question 22 /!\**

**20. List 1 to 3 ways that the congregation supports the programmes to address overweight or obesity among adolescents (we only want to know about existing programmes)**

^1.^_____________________________________________________________________

^2.^ _____________________________________________________________________

^3.^ _____________________________________________________________________

**21. List some of the barriers to programmes addressing adolescent overweight or obesity in your congregation:**

^1.^_____________________________________________________________________

^2.^ _____________________________________________________________________

^3.^ _____________________________________________________________________

**22. Do you think overweight or obesity is a problem for adolescents?**

^1.^ 🞎 Not at all

^2.^ 🞎 Somewhat

^3.^ 🞎 Very much

**23. Do other people in your congregation think overweight or obesity is a problem for adolescents**

^1.^ 🞎 Not at all ^2.^ 🞎 Somewhat ^3.^ 🞎 Very much ^4.^ 🞎 Don’t know

**24. Do religious leaders know a lot about overweight or obesity?**

^1.^ 🞎 Not at all

^2.^ 🞎 Somewhat

^3.^ 🞎 Very much

**25. List 1 to 3 types of information that are available in your congregation regarding overweight or obesity:**

^1.^_____________________________________________________________________

^2.^ _____________________________________________________________________

^3.^ _____________________________________________________________________

**26. Is any of this information for adolescents?**

^1.^ 🞎 Yes ^2.^ 🞎 No

**27. List 1 to 3 types of local sources of information on overweight or obesity in your congregation?**

^1.^_____________________________________________________________________

^2.^ _____________________________________________________________________

^3.^ _____________________________________________________________________

**28. Is any of this information for adolescents?**

^1.^ 🞎 Yes ^2.^ 🞎 No

**29. List 1 to 3 ways people obtain information about overweight or obesity problems in your congregation**

^1.^_____________________________________________________________________

^2.^ _____________________________________________________________________

^3.^ _____________________________________________________________________

**30. Is any of this information for adolescents?**

^1.^ 🞎 Yes ^2.^ 🞎 No

**31. List 1 to 3 people to whom in your Church an overweight or obese adolescent would turn to first for help?**

^1.^_____________________________________________________________________

^2.^ _____________________________________________________________________

^3.^ _____________________________________________________________________

**32**. **Why do you think they would turn to these people?**

^1.^_____________________________________________________________________

^2.^ _____________________________________________________________________

^3.^ _____________________________________________________________________

**33a.What is the congregation’s attitude about people volunteering time to address obesity**

^1.^ 🞎 Supportive ^3.^ 🞎 Not supportive

^2.^ 🞎 Somewhat supportive ^4.^ 🞎 Don’t know

**33b. What is the congregation’s attitude about people making financial donations**

^1.^ 🞎 Supportive ^3.^ 🞎 Not supportive

^2.^ 🞎 Somewhat supportive ^4.^ 🞎 Don’t know

**33c. What is the congregation’s attitude about people providing space**

^1.^ 🞎 Supportive ^3.^ 🞎 Not supportive

^2.^ 🞎 Somewhat supportive ^4.^ 🞎 Don’t know

**34. List up to 3 proposed programmes/action plans that have been submitted for funding that address overweight or obesity in your Church?**

^1.^_____________________________________________________________________

^2.^ _____________________________________________________________________

^3.^ _____________________________________________________________________

**35. Do you know if there is any evaluation of programmes that are in place to address overweight or obesity?**

^1.^ 🞎 Yes

^2.^ 🞎 No

^3.^ 🞎 Don’t know

**/!\ If no or don’t know, then the survey is finished /!\**

**36. If yes, how effective is the evaluation of these programmes (with 1 being “not at all” and 10 being “very good?”)**

1 🞎 2 🞎 3 🞎 4 🞎 5 🞎 6 🞎 7 🞎 8 🞎 9 🞎 10 🞎

Not at all Average Very good

☹ 😐 ☺

**37. Are the evaluation results being used to make changes in programmes, activities, or policies or to start new ones?**

^1.^ 🞎 Yes ^2.^ 🞎 No

If yes, please explain______________________________________________________

**Thank you very much for completing this survey**
