# Supplementary material for: Community readiness for adolescents’ overweight and obesity prevention is low in urban South Africa: a case study
Source: BMC Public Health. 2016 Aug 11;16:763. doi: 10.1186/s12889-016-3451-9 (PMC4982405; doi:10.1186/s12889-016-3451-9)
Supplement: Additional file 2: — Focus group interview guide. (DOCX 226 kb) [file 12889_2016_3451_MOESM2_ESM.docx]

**Appendix 2.** Focus group interview guide

| **Themes** | **Questions** |
| --- | --- |
| **Issues in the community** | What is the most important issue of concern that comes to mind? |
|  | What are the second and third most important issues that come to mind? |
|  | Why is X the most important issue for adolescents in the congregation?  Do you think these concerns in your congregation reflect the greater community/area where you live? Tell us why or why not. |
| **Body image perceptions (silhouettes)** | 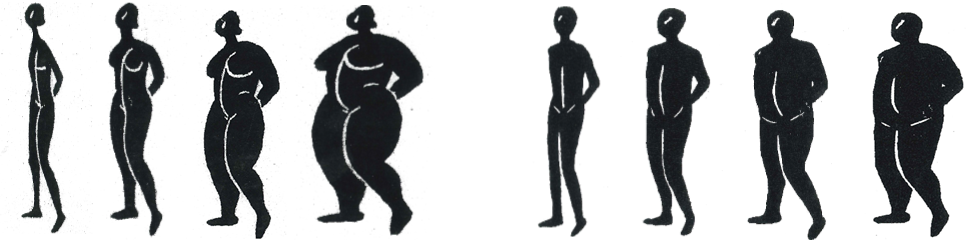 |
| **Obesity definition** | Could you define obesity? Can you describe what obesity is? |
|  | When you think about the word obesity, what comes to mind?  Do you use other words to describe obesity? |
|  | How are obese people perceived in your congregation?  Are all obese people perceived in the same way? |
|  | Why do you think people are obese?  What are the causes of obesity in your congregation? |
|  | What makes someone obese? What foods might contribute to obesity?  What are factors other than food that might make somebody obese? |
|  | Can you tell us about any risks or benefits associated with being obese? |
| **Unhealthy dietary habits** | Could you tell us something about unhealthy dietary habits that you are aware of in your community? |
|  | What are healthy and less healthy foods for adolescents? |
|  | What foods are healthy?  Do adolescents commonly consume these foods? |
|  | What foods are unhealthy?  Do adolescents commonly consume these foods? |
|  | What are the risks of adopting unhealthy dietary habits? |

**Appendix 2.** Focus group interview guide (continued)

| **Themes** | **Questions** |
| --- | --- |
| **Role of**  **the Church** | What is the role of the Church in adolescents’ health? |
|  | What is the role of the Church in relation to adolescent obesity? What role does your congregation play in adolescents’ knowledge about obesity? |
|  | What is the role of the Church in relation to adolescent dietary habits? |
|  | Tell me anything that your congregation does to encourage adolescents to eat healthy or less healthy foods? What makes the congregation do these things to encourage these eating patterns? |
|  | What is the role of the Church in relation to encouraging adolescents to take part in physical activity (exercise)? |
|  | What types of activities are available in your congregation for adolescent health? From where do you think that the adolescents in your congregation receive information about health? |
| **Relationship between the religious leaders and adolescents** | Who do you think the adolescents in your congregation perceive to be a role model? Which leaders in the Church are seen as the biggest role models for adolescents? |
| **Resources** | What resources do leaders in the community have to help adolescents maintain healthy weights? |
|  | What are some of the challenges leaders in the Church face with regard to adolescent obesity? What additional resources might make it possible for the Church to work with adolescents regarding obesity? |
|  | What are some opportunities leaders in the Church have for making an effect on adolescent obesity in the congregation? What are some of the constraints that would make this more difficult? |
