# Supplementary material for: Community readiness for adolescents’ overweight and obesity prevention is low in urban South Africa: a case study
Source: BMC Public Health. 2016 Aug 11;16:763. doi: 10.1186/s12889-016-3451-9 (PMC4982405; doi:10.1186/s12889-016-3451-9)
Supplement: Additional file 3: — Results of the thematic analysis. (DOCX 17 kb) [file 12889_2016_3451_MOESM3_ESM.docx]

**Appendix 3**. Results of the thematic analyses

| **Themes** | **Sub-themes** | **Codes** |
| --- | --- | --- |
| **Community issues** | *Health issues* | STDs, HIV |
|  |  | Teenage pregnancies |
|  |  | Obesity |
|  |  | Unhealthy dietary habits, inadequate food for families |
|  |  | Access to health care and information |
|  | *Social issues* | Peer pressure, image, stress, depression |
|  |  | Technology (radio, tv, social networks) |
|  |  | Change in family structure, single parenthood, dysfunctional families |
|  |  | Loss of identity |
|  |  | Environmental change |
|  |  | Cultural change |
|  |  | Discipline, delinquency, unruliness |
|  |  | Shifts in lifestyle, working patterns |
|  |  | Unemployment |
|  |  | Lack of development activities, empowerment programs |
|  |  | Hopelessness, uncertainty about future |
|  |  | Inequalities, poverty |
|  |  | Drugs, alcohol |
|  | *Safety issues* | Violence |
| **Obesity** | *Definition* |  |
|  | *Causes* | Genetic |
|  |  | Knowledge |
|  |  | Laziness |
|  |  | Medication |
|  |  | Disease |
|  |  | Affordability |
|  |  | Diet, lack of fasting, alcohol |
|  |  | Physical activity, lack of resources for young people to embark on exercise |
|  |  | Depression, stress |
|  |  | Lack of educational programs |
|  |  | Parental influence |
|  | *Consequences* | Early death, prone to diseases, serious health problems |
|  |  | CVDs, high cholesterol |
|  |  | Hypertension |
|  |  | Diabetes |
|  |  | Low self-esteem, target of ridicule, stereotyping, stigmatization, social outcast |
|  |  | Mobility reduction, decrease in concentration levels |

**Appendix 3.** Results of the thematic analyses (continued)

| **Themes** | **Sub-themes** | **Codes** |
| --- | --- | --- |
| **Unhealthy dietary patterns** | *Definition of healthy foods* | Non fatty meat, fruit and vegetables, white meat, unrefined maize-meal, mopani worms, organic foods, freshly cooked food, food fresh out of the soil, pap |
|  | *Definition of unhealthy foods and unhealthy dietary habits (meal structure, portion sizes)* | Refined maize-meal, full cream milk, potatoes, chocolate, fat cakes, eggs, mango-atchar, fried potato chips, bunny chows, red meat, fast foods and take-away, sweets, noodles, unhealthy ways of preparing foods, sauces, Huge portion sizes, light meals in the morning and heavy meals in the evening, preference for easy to prepare foods, easily accessible, high consumption of fizzy drinks and processed food |
|  | *Definition of "eating well"* | Having a balanced meal regularly, take away food once in a while, balanced breakfast, lunch and supper in controlled portions |
|  | *Reasons for adopting unhealthy dietary habits* | cheaper, quick, convenient, habit, taste, poverty, taste over nutrient content and over substance issue, ignorance, time factor |
|  | *Cultural change* |  |
| **Physical activity patterns** | *Causes* | Cost, safety, technology, modernisation |
| **Body image perceptions** | *Sex differences* |  |
|  | *Age differences* |  |
|  | *Size differences* |  |
|  | *Urban vs. Rural differences* |  |
|  | *Cultural change* |  |
| **Church's role** | *Role of the church in adolescent life* | Counselling and teaching, influential role, spiritual influence, listening and guiding |
|  | *Role of the church in adolescent health (obesity, diet, etc.)* |  |
|  | *Relationship between leaders and adolescents* | Role models |
|  | *Resources* | Knowledge |
|  |  | Time |
|  |  | Facilities  Money |
|  |  | Bible writings |
|  |  | Church's members (adolescents, people attending services) |
|  | *Challenges* | Time, knowledge, lack of speakers on health issues, risk of stigmatisation. |
|  | *Opportunities* | Networks, inventory of members' skills, role models |
| **Key informants in community** | *Religious bodies, families, social workers, schooling system* |  |
